# Cancer detection using human papillomavirus self‐sampling targeting long‐term non‐attenders in an organized cervical screening program

**DOI:** 10.1002/ijc.70321

**Published:** 2025-12-29

**Authors:** K. Miriam Elfström, Maria Hortlund, Daniel Öhman, Joakim Dillner

**Affiliations:** ^1^ Center for Cervical Cancer Elimination Karolinska Institutet and Karolinska University Hospital Stockholm Sweden; ^2^ Unit for Cancer Screening Regional Cancer Center of Stockholm‐Gotland Stockholm Sweden; ^3^ Present address: NovoNordisk A/S Søborg Denmark

**Keywords:** cancer, HPV, human papillomavirus, screening, self‐sampling

## Abstract

Self‐sampling for human papillomavirus (HPV) is an established strategy to increase participation in cervical screening. We previously reported a randomized trial targeting women who had not attended screening after >10 invitations, where sending of self‐sampling kits resulted in a 19% attendance and a positive predictive value (PPV) for high grade lesions (HSIL+) of 40%, despite no triaging after the HPV test. Because of the striking results, the intervention was extended to all women resident in Stockholm County, Sweden, in the years 2019/20, who had not attended screening >10 years (*N* = 42,409). Participation was 35.6% and 11.6% of the participating women were HPV‐positive. Among these, there were 43 cases of invasive cervical cancer and 319 cases of high‐grade lesions. The PPV was particularly high for HPV16/18 positive women (12% for invasive cancer and 59% for HSIL). In summary, participation with HPV self‐sampling among long‐term non‐attenders in the real‐life program was considerably higher than in the research setting and the high yield of HSIL+ implied high effectiveness.

## What's New?

The strategy to offer self‐sampling for human papillomavirus testing to increase cervical cancer screening attendance among long‐term non‐attenders has been mostly validated in randomized trials. This real‐life implementation of self‐sampling for long‐term non‐attenders to the routine screening program in the capital region of Sweden yielded the high participation of 35.6%, with 11.6% of the participating women being HPV‐positive. The results showed high positive predictive values for high‐grade lesions and invasive cervical cancer among the referred women. Women's participation was considerably higher than that in the research setting, and the high yield of high‐grade lesions implied high effectiveness.

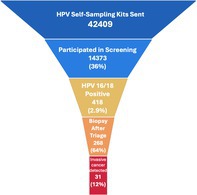

**Abbreviations**
HPV: human papillomavirus
HSIL: high grade lesion
ICC: invasive cervical cancer
NKCx: The Swedish National Cervical Screening Registry
PPV: positive predictive value
RCT: randomized clinical trial
WHO: World Health Organization

## 1 INTRODUCTION

Cervical screening using a high‐performance human papillomavirus (HPV) test is one of the pillars of the World Health Organization (WHO) strategy for global elimination of cervical cancer.^1^ In contrast to the previously used cervical screening method (cytology), the sample for HPV testing can be taken by the woman herself allowing for greatly simplified screening program logistics.^2^ When self‐samples are analyzed for HPV using polymerase chain reaction, the sensitivity is not inferior to clinician collected samples.^2^ Self‐sampling is commonly used to increase population coverage of screening, particularly in projects targeting under‐screened and hard‐to‐reach women.^3^, ^4^

Although the strategy to use self‐sampling for improved attendance among hard‐to‐reach women has been extensively validated in randomized trials,^5^ results from studies in the research setting are not always generalizable to the real‐life setting. For example, the randomized clinical trial (RCT) by Polman et al. had to write to >187,000 women to enroll 16,000 trial participants willing to try the self‐sampling.^5^

We have previously reported on a randomized healthcare policy trial, where the screening program in Stockholm, Sweden randomized long‐term non‐attenders into four arms, one of which was directly sent a self‐sampling kit.^6^ As the attendance rate was greatly increased (11‐fold) and the PPV for HSIL+ among HPV‐positive women (also without triaging) was very high, we considered it an ethical obligation to offer the self‐sampling to all non‐attenders (also those previously randomized to inferior strategies). We report the participation rate and the PPV for HSIL+ when the intervention was offered not as a randomized trial, but as a screening policy targeting all long‐term non‐attenders.

## 2 METHODS

**FIGURE 1:**
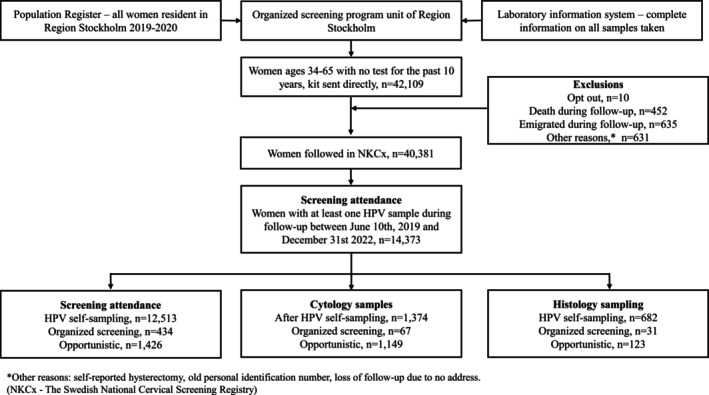
Flowchart of the study population including cytology and histology follow‐up among women participating with an HPV sample after invitation. ^a^Other reasons: Self‐reported hysterectomy, old personal identification number, loss of follow‐up due to no address (NKCx—The Swedish National Cervical Screening Registry).

To define the target population, we first identified all women resident in the Stockholm/Gotland region in the target group for screening (ages 23–65 in the region). The Swedish screening program has recommended screening for women ages 23–70 since 2015, but the upper age limit was extended only gradually in the region. Women were included who either (1) had received one or several summons for screening but had no sample taken in the region for >10 years or (2) had no sample taken in spite of being invited >11 years ago. All samples taken were considered (not only organized samples) and for technical reasons, all gynecological samples recorded (topology code T8*) were considered. This identified 42,409 women to whom the self‐sampling kit was sent in 2019/2020 (Figure 1). There were 10 women who opted out of the program, 452 who died, 635 who emigrated and 631 who could not be followed up for other reasons (e.g., self‐reported hysterectomy or incorrect personal identity number). Finally, 40,381 women could be followed up using the Swedish National Cervical Screening Registry, NKCx.se (Figure 1). Eligible women were sent the STI (sexually transmitted infections) sample collection kit (Roche diagnostics) and the returned samples were tested with the Cobas 4800 HPV testing platform (Roche diagnostics) that tests for HPV16 and HPV18 in separate channels and 12 other HPV types (HPV31/33/35/39/45/51/52/56/58/59/66/68) in a third channel. In this paper, “HPV‐positive” refers to positivity to any of the 14 HPV types detected by the Cobas test.

All HPV screening attendance was assessed, including returning the self‐sampling kit, attendance at a midwife clinic, and all HPV tests performed outside of the program (e.g., in private gynecology clinics) as these also report to the screening registry.

In the randomized trial, HPV positive women had been referred to colposcopy directly without triaging. For the cohort in the present report, HPV positive women were triaged using cytology before referral to colposcopy as there was uncertainty regarding the capacity of colposcopy services to manage all HPV‐positive women in the cohort. Women were followed in the registry for the outcomes of subsequent high grade squamous intraepithelial neoplasia or worse (HSIL+) and invasive cervical cancer (ICC) through December 31, 2022. Sweden has extensive quality checking of cancer registry data, resulting in rather long delays before publication (>1 year). Descriptive statistics were calculated to examine participation, HPV prevalence, and cytology and histopathology outcomes by screening modality and HPV type. The positive predictive value (PPV) for HSIL+ and ICC was calculated by screening modality and HPV screening results.

## 3 RESULTS

The mean age of the cohort was 50 years and there were no women below 34 years of age (screening starts at age 23 and non‐attendance for at least 11 years was a requirement) (Table 1). The screening programs in Sweden are regionally organized and do not have access to tests taken outside their region. For evaluation of this study, we linked to the National Cervical Screening Registry and found that 5.3% of the women who had not participated in Stockholm did have a cervical screening test taken in the preceding 10 years, most likely representing cervical screening tests taken in other parts of Sweden (Table 1). For 27% of the target group, there was no record of screening on file at all and for the remaining 67%, there were screening tests on file, but taken more than 10 years ago (Table 1).

**TABLE 1:** Characteristics of the study participants.

Characteristic	Participation (%)	HPV positivity (%)	HSIL+ (%)	Total (%)
Age category^a^				
Age 34–50 years	7640 (39.8)	991 (13.0)	221 (2.9)	19,201 (47.5)
Age 51–65 years	6733 (31.8)	670 (10.0)	98 (1.5)	21,180 (52.5)
Time since last cervical sampling				
0–10 year	1171 (55.1)	159 (13.6)	26 (2.2)	2124 (5.3)
>10 years	9976 (36.6)	1132 (11.3)	212 (2.1)	27,228 (67.4)
No previous record	3226 (29.3)	370 (11.5)	81 (2.5)	11,029 (27.3)
Total				40381^b^

^a^ Mean age in years 50.2 (SD 8.85).

^b^ An additional 1728 women were excluded because of opt out (10), death (452), emigration (635), and other reasons (631, including self‐reported hysterectomy, old personal identification number, loss of follow‐up due to no address).

Overall, participation following invitation to self‐sampling in the cohort was 35.6% (Table 2), 31.0% returned a self‐sampling kit sent, and 1.1% participated at a midwife clinic (representing routine sampling modality in the organized screening program). Interestingly, 3.5% of the women in the cohort participated through non‐organized testing (e.g., in a private gynecology clinic) even though the women in the targeted cohort had no previous attendance in either organized or non‐organized testing. The HPV prevalence was 11.6%, with little variation depending on who had taken the sample (Table 2). The highly oncogenic HPV types HPV16/18 were found in only 2.9% of the tested women.

**TABLE 2:** Participation, HPV, cytology, and histology results by sampling mode in the follow‐up time June 10, 2019 to December 31, 2022.

Results	Self‐sampling, *n* (%)	Clinical collected sampling		Total, *n* (%)
		Organized screening, *n* (%)	Opportunistic, *n* (%)	
Participation^a^	12,513 (31.0)	434 (1.1)	1426 (3.5)	14,373/40,381 (35.6)
High risk HPV positive	1440 (11.5)	53 (12.2)	168 (11.8)	1661 (11.6)
HPV 16/18 positive	343 (2.7)	20 (4.6)	55 (3.9)	418 (2.9)
HPV 16 positive	275 (2.2)	16 (3.7)	43 (3.0)	334 (2.3)
HPV 18 positive	71 (0.6)	4 (0.9)	12 (0.8)	87 (0.6)
Cytology results^b^	1374 (11.0)	67 (15.4)	1149 (80.6)	2590/14,373 (18.0)
HSIL or worse in cytology^c^	214 (15.6)	17 (25.4)	62 (5.4)	293 (11.3)
Histopathology results^b^	682 (5.5)	31 (7.1)	123 (8.6)	836/14,373 (5.8)
High risk HPV positive^d^	599 (87.8)	28 (90.3)	83 (67.5)	710 (84.9)
HPV 16/18 positive^d^	222 (32.6)	12 (38.7)	34 (27.6)	268 (32.1)
HPV 16 positive^d^	178 (26.1)	10 (32.3)	27 (22.0)	215 (25.7)
HPV 18 positive^d^	44 (6.5)	2 (6.5)	7 (5.7)	53 (6.3)
Histopathology results—HSIL+^d^	256 (37.5)	18 (58.1)	45 (36.6)	319/836 (38.2)
High risk HPV positive	254 (37.2)	18 (58.1)	44 (35.8)	316 (37.8)
HPV 16/18 positive	123 (18.0)	8 (25.8)	27 (22.0)	158 (18.9)
HPV 16 positive	105 (15.4)	7 (22.6)	21 (17.1)	133 (15.9)
HPV 18 positive	18 (2.6)	1 (3.2)	6 (4.9)	25 (3.0)
Histopathology results—Invasive cervical cancer^d^	29 (4.3)	4 (12.9)	10 (8.1)	43/836 (5.1)
High risk HPV positive	28 (4.1)	4 (12.9)	9 (7.3)	41 (4.9)
HPV 16/18 positive	20 (2.9)	2 (6.5)	9 (7.3)	31 (3.7)
HPV 16 positive	16 (2.3)	2 (6.5)	7 (5.7)	25 (3.0)
HPV 18 positive	4 (0.6)	—	2 (1.6)	6 (0.7)

^a^ Among all invited women.

^b^ Among all participating women.

^c^ Among all women with a cytology result.

^d^ Among all women with a histology result—HSIL, AIS, invasive cervical cancer.

The proportion of women who, after testing HPV positive in self‐sampling, attended a follow‐up visit for cytology was 1374/1440 (95.4%) or 11.0% of all women participating with self‐sampling. The proportion of women who had a cytology among those with a non‐organized HPV test was higher than the number of positive women as it is common in private gynecology practice to order co‐testing for both HPV and cytology.

There were 836 women in the cohort who had a cervical histopathology taken, including 319 women with HSIL in histopathology and 43 cases of ICC (Table 2). About a third of the histopathologies taken outside the program were taken among HPV‐negative women (Table 2), presumably because double testing with both cytology and HPV is common among private practitioners. There were two cervical cancers and three cases of HSIL among the HPV‐negative women in the cohort (not shown). A majority of both HSIL and cervical cancers were found among the women with the high oncogenicity HPV types 16/18, in spite of the fact that they constituted only a fourth of all HPV‐positive women. HPV16 was particularly dominant, and responsible for 25 cases of ICC and 133 cases of HSIL (Table 2).

The PPV to detect HSIL among the HPV‐positive women was 44.5%, again highest for the women with HPV16 (61.9%) (Table 3). PPV for ICC was 5.8% among HPV‐positive women and 11.6% among HPV16‐positive women. HPV18 had the typical pattern of being relatively more important in invasive cancer (PPV 11.3%, similar to HPV 16) than in HSIL (47.2%, much lower than the PPV of HPV 16 for HSIL) (Table 3).

**TABLE 3:** Positive predictive value (PPV) for HSIL and invasive cervical cancer (ICC) by HPV status and sampling mode.

PPV	Self‐sampling, *n* (%)	Total^a^, *n* (%)
PPV to detect HSIL		
High risk HPV positive	42.4% (254/599)	44.5% (316/710)
HPV 16/18 positive	55.4% (123/222)	59.0% (158/268)
HPV 16 positive	59.0% (105/178)	61.9% (133/215)
HPV 18 positive	40.9% (18/44)	47.2% (25/53)
PPV to detect ICC		
High risk HPV positive	4.7% (28/599)	5.8% (41/710)
HPV 16/18 positive	9.0% (20/222)	11.6% (31/268)
HPV 16 positive	9.0% (16/178)	11.6% (25/215)
HPV 18 positive	9.1% (4/44)	11.3% (6/53)

^a^ Cervical samples taken by healthcare personnel after the mailing of the self‐sampling kit.

## 4 DISCUSSION

We report that the addition of organized self‐sampling for HPV targeting long‐term non‐attending women to organized screening resulted in both high attendance rates, high PPVs for HSIL among referred women, and a substantial yield of detected HSIL and invasive cancer.

Strengths of our study are that it evaluates a population‐based effort performed entirely within the routine screening program and has comprehensive follow‐up using a nationwide complete registry, implying generalizability and reliability. Furthermore, the large size of the study enabled us to report on the yield of ICC with precision, even estimating the HPV type‐specific PPVs for invasive cancer detection. As the study was a one‐time effort with the sending of self‐sampling materials, biases by calendar time trends were unlikely. There was no non‐organized self‐sampling in Sweden, facilitating interpretation.

Weaknesses include that the intervention for ethical reasons targeted all eligible women, resulting in that we can only compare attendance and yield with previous results where a randomized control group was included^6^ or with the whole country of Sweden. For example, our participation rate of 36% is strikingly higher than in the control group with standard invitations to sampling by healthcare personnel (1.3%) and also much higher than the similar intervention in our RCT (18%). Comparison to the total amounts of cervical samples analyzed in Sweden also suggests that the intervention was effective. Overall, there were >1.7 million invitations to screening sent and >1 million cervical tests taken in Sweden in 2019 (www.nkcx.se, accessed on September 2, 2025). There are annually about 550 cases of cervical cancer in Sweden, with 22% of these (about 120 cases) being screen‐detected.^7^ Thus, although we do not have an exact comparison group, we can conclude from the national statistics that diagnosis of 43 invasive cancers after mailing just 42,000 kits and testing about 14,000 samples suggests that the intervention was effective. Although cervical screening primarily aims to prevent cervical cancer by detection and treatment of precursor lesions, improvement of the prognosis of the cancer by screen‐detection rather than symptom‐detection is also an important goal of the program.^8^

A one‐time effort such as ours is readily evaluated by registry‐based follow‐up, whereas for an ongoing program with multiple invitations and reminders over time it is more difficult to discern which samples and biopsies resulted from which invitation. For evaluations comparing only participation rates, it is advisable to focus on the participation directly linked to the invitation.^9^ However, sending self‐sampling kits can induce an increase in attendance to physical screening visits^6^ resulting in that evaluations focusing only on the yield with the self‐samples will underestimate the effect of the intervention.

This study suggests that real‐life program use, rather than use in research studies, should be added to the known factors that increase attendance. Possible reasons for this are that research studies may be perceived as testing new things of uncertain value and that there may be stronger motivation to participate in real programs.

The most effective strategy for self‐sampling is to distribute and collect, followed by direct send, and lastly by invitation to order.^2^ Invitation to order is still a more effective strategy to reach long‐term non‐attenders than invitations to a physical visit (Table 4).

**TABLE 4:** Context and outcomes.

Context and outcomes	This study	The routine screening program: invitation to sampling visit	Original LTNA trial^a^—direct send kit	Original LTNA trial^a^—invitation to order kit	Original LTNA trial^a^—invitation to sampling visit	Nationwide LTNA trial^b^
Setting	Routine screening	Routine screening	Research	Research	Research	Research
Population	All LTNA >10 years in Stockholm region	Everyone targeted for screening in Sweden	Random sample of LTNA >10 years in Stockholm region	Random sample of LTNA >10 years in Stockholm region	Random sample of LTNA >10 years in Stockholm region	LTNA or non‐attendance to follow‐up in whole of Sweden
Triage visit with cytology	Yes	Yes	No	No	No	No
Strategy previously tried	No	Yes	No	No	Yes	Yes
Target population	42,409	2,950,711	2000	2000	2000	28,689
Number of women invited	42,409	541,093^c^	2000	2000	2000	28,689
Number participated (%)	14,373 (35.6%)	340,889 (63%)^c^	374 (18.7%)	213 (10.6%)	34 (1.7%)	2853 (9.9%)
Number screen‐positive (% among tested)	1661 (11.6%)	21,118/239,608 (8.8%) (HPV tests). 12,170/115183 (19.6%) (cytologies)^d^	48 (12.8%)	26 (12.2%)	3 (9%)	417 (14.6%)
Attending triage visit (proportion of screen‐positives)	1374/1440 (95.4%)^e^	NA^e^	NA^e^	NA^e^	NA^e^	NA
Total number of biopsies (% of target population)	836 (2.0%)	25,614 (0.87%)^f^	36 (1.8%)	23 (1.2%)	1 (0.05%)	202 (0.7%)
Total number of CIN2+ (% of target population)	319 (0.75%)	6909 (0.23%)^f^	17 (0.85%)	5 (0.25%)	1 (0.05%)	44 (0.15%)
Total number of screen‐detected cancers (% of target population)	43 (0.10%)	120 (0.004%)^f^	NA^g^	NA^g^	NA^g^	4 (0.014%)

^a^ The original trial is from Elfström et al.^6^

^b^ This study^10^ used only invitation to order a kit (no direct send).

^c^ Recently screened women are not invited, invitations are only issued after a screening interval has passed. This number does not count reminder invitations. If including reminder invitations, >1.7 million invitations per year are issued. The number of tests taken is the tests taken as a result of a primary invitation. If also counting tests taken after reminder invitations or tests taken without invitation, there are >1 million tests/year.

^d^ Total number of tests is somewhat higher than the number of visits, because of some co‐testing with both HPV and cytology. The high number of positive cytologies is because cytology was mostly used among women below 30 years of age.

^e^ There were only 1440 screen‐positive women referred after self‐sampling. Some women who received the kit attended physical visits instead. If attending for symptoms, there would have been co‐testing with both HPV and cytology. Attendance to triaging after self‐sampling can thus only be calculated for the women who actually used the self‐sampling option. In the routine program, triaging is done on the same sample and does not require a new visit. In the original trial, women were referred without triaging.

^f^ Whereas the counts on number of biopsies in the other columns are derived from follow‐up of the cohort in the column, this is all the biopsies in a subsequent calendar year in the target population (could have resulted from HPV testing or cytologies also in previous years).

^g^ Because of small numbers, invasive cancers were included in CIN2+ but not separately recorded.

The strongest factors affecting attendance appear to be if the same strategy has been used before. A striking example of this is presented in the comparative Table 4, where standard first invitations to a physical visit to women due for the next round of screening had a 63% attendance, whereas the same invitation to women who had unsuccessfully been invited 10 times before (long‐term non‐attenders) had a 1.7% attendance. Similarly, a study that used invitation to order a self‐sampling kit found a certain attendance at first invitation, a decreased attendance at the second invitation, and no attendance at all at the third invitation.^10^

An additional factor affecting attendance is if there is a triaging visit. In previous trials, we have referred HPV‐positive women directly to colposcopic evaluation without a new visit for a cytology triage test as we found it to be unnecessary when targeting women who were already at high risk. In the present study, we did use an extra triage visit before referral to colposcopy. As attendance at the triage visit was high (>95%), the overall yield of the intervention suffered only marginally.

About 7.5% of cervical cancers are negative for HPV,^11^ with HPV‐negative cancers primarily being found in late‐stage cancers found among never attenders. It was therefore a concern whether there may be an enrichment of HPV‐negative cancers among non‐attending women, who would then not be detected by the HPV test. The high yield of cancers among the HPV positives and limited number of HPV‐negative cancers (two cancers among 126 biopsies taken among HPV‐negative women as compared to 25 cancers among 215 biopsies taken among HPV16‐positive women) suggests that HPV self‐sampling was nevertheless an appropriate strategy to offer to long‐term non‐attending women.

In 2021, preliminary data on attendance from this study was available when the screening program in Stockholm County decided to switch to self‐sampling for HPV as the primary screening strategy not only for long‐term non‐attending women, but all women invited to routine screening ages 26–70.^9^ The result was a strong increase in population test coverage in just 1 year.^9^ Nationally, self‐sampling can now be used as an alternative to clinician collected samples in the routine program for women regardless of their screening history. The high PPVs seen have resulted in that the Swedish guidelines prescribe that in long‐term non‐attender groups, HPV‐positive women should be referred directly to colposcopy without triaging. Similarly, Sweden has also adopted the practice already used in, for example, USA and Australia that all HPV16/18‐positive women should be referred to colposcopy (https://kunskapsbanken.cancercentrum.se/diagnoser/livmoderhalscancerprevention/vardprogram/).

In summary, the present evaluation of introducing routine HPV self‐sampling for long‐term non‐attending women demonstrated that the strategy results in higher participation than found in previous studies in the research setting and has underpinned the continued use of this strategy.

## AUTHOR CONTRIBUTIONS

**K. Miriam Elfström:** Investigation; writing – review and editing; project administration; supervision. **Maria Hortlund:** Formal analysis; investigation; visualization; writing – review and editing. **Daniel Öhman:** Data curation; validation; project administration; writing – review and editing. **Joakim Dillner:** Conceptualization; methodology; resources; supervision; writing – original draft.

## FUNDING INFORMATION

Funded by the Swedish Association of Local Authorities and Regions and the Stockholm County Council. The original randomized trial was funded by the Nordic Information for Action eScience Center (NIASC), a Nordic Center of Excellence in eScience funded by NordForsk (Project no. 62721).

## CONFLICT OF INTEREST STATEMENT

The authors have no conflicts of interest related to the content of this work.

## ETHICS STATEMENT

The study was granted ethical approval by the Stockholm Regional Ethical Review Board (DNR 2015/1705‐31/1). The original trial^6^ is registered at clinicaltrials.gov under NCT02750124. Informed consent for participation in the current study is obtained through an opt‐out procedure. Participants are provided with written information in the invitation to screening about the personal data registration and program evaluation and are given the opportunity to decline participation.

## Data Availability

The data that support the findings of this study are available from the corresponding author upon reasonable request.
